# Super‐recognisers in Action: Evidence from Face‐matching and Face Memory Tasks

**DOI:** 10.1002/acp.3170

**Published:** 2015-10-20

**Authors:** Anna K. Bobak, Peter J. B. Hancock, Sarah Bate

**Affiliations:** ^1^ Department of Psychology Bournemouth University Dorset UK; ^2^ Psychology, School of Natural Sciences University of Stirling Stirling UK

## Abstract

Individuals employed in forensic or security settings are often required to compare faces of ID holders to document photographs, or to recognise the faces of suspects in closed‐circuit television footage. It has long been established that both tasks produce a high error rate amongst typical perceivers. This study sought to determine the performance of individuals with exceptionally good face memory (‘super‐recognisers’) on applied facial identity matching and memory tasks. In experiment 1, super‐recognisers were significantly better than controls when matching target faces to simultaneously presented line‐ups. In experiment 2, super‐recognisers were also better at recognising faces from video footage. These findings suggest that super‐recognisers are more accurate at face matching and face memory tasks than typical perceivers, and they could be valuable expert employees in national security and forensic settings. © 2015 The Authors Applied Cognitive Psychology Published by John Wiley & Sons Ltd.

Facial perception is the most reliable means of accessing a person's identity without the use of automated technology, such as iris or fingerprint analysis. Yet, there are large individual differences in the ability to recognise (Bowles et al., 2009; Russell, Duchaine & Nakayama, 2009) and perceive (Megreya & Bindemann, 2013; Megreya & Burton, 2006) faces, and particular difficulties are associated with the processing of unfamiliar facial stimuli (see Hancock, Bruce & Burton, 2000 for a review). For instance, over 80 trials, Bruce et al. (1999) asked participants to select a face from an array of 10 that matched the identity of a target face. Stimuli presented within the arrays were photographic images, whereas the target faces were still images acquired from video footage. Despite optimal viewing conditions and no time constraint, the accuracy of face matching was merely 70%. The photographs and videos used in the study were even taken on the same day, enabling the use of identity cues from external features, such as hairstyle. It is possible that, if the images and videos had been taken on different days, accuracy would have been even lower. Indeed, Megreya, Sanford and Burton (2013) reported that, when photographs were taken months apart, recognition accuracy decreased by approximately 15%.

Most research examining face matching and recognition uses high‐quality images taken from purpose‐built face databases. However, real‐life situations often require the matching or recognition of faces from closed‐circuit television (CCTV) — systems that are widely used in most developed countries but provide images of considerably poorer quality than high‐resolution digital photographs. With the first large‐scale installation of public cameras occurring in Bournemouth in 1985, there are currently approximately 4 million devices in the UK (Security Newsdesk, 2013) and 30 million in the USA (Davis & Valentine, 2009). Typically, there are two situations in which CCTV evidence can be useful. First, a person displayed in CCTV footage that is released to the public can be recognised by someone familiar to them, such as a family member, colleague or neighbour. Second, CCTV evidence can be used in a court of law to match the identity of a defendant in the dock.

It has been widely established that, while identification of familiar faces from CCTV footage is highly reliable and accurate (Burton, 2013; Burton, Wilson, Cowan & Bruce, 1999), the task of identifying or even matching unfamiliar faces is characterised by a high rate of error (Bruce et al., 1999; Bruce, Henderson, Newman & Burton, 2001; Burton et al., 1999). Indeed, Burton et al. (1999) demonstrated that the recognition of familiar faces, even from poor‐quality CCTV, is fast and effortless. Conversely, the identification of unfamiliar faces in the same task was close to chance. Similarly, Hill and Bruce (1996) studied unfamiliar face matching across different viewpoints and lighting conditions. They found that participants were very accurate when pose and lighting position were matched in both pictures (e.g. frontal view and top lighting) but dropped dramatically when pose or lighting type differed between pictures. These results were similar to those reported by Bruce et al. (1999), who found that a change in viewpoint was particularly detrimental in unfamiliar face matching, where participants mainly used external features to make their judgements.

In everyday situations, person‐to‐ID matching is commonly encountered in various contexts. Kemp, Towell and Pike (1997) investigated fraud detection from credit cards in a group of experienced supermarket cashiers. The authors reported that in the trials where identities did not match but the photograph resembled the card bearer, cashiers only correctly rejected the identities in 36% of cases (a 64% error rate). What is more, in the trials where the bearers did not resemble the photographs, the error rate was still high at 34%. This high error rate occurred despite the fact that the cashiers were aware of the study and presumably motivated to perform the task well.

In forensic settings such as border control or crime investigation, successful matching of identities from a photograph to a real person is a matter of national security. In a recent report, White, Kemp, Jenkins, Matheson and Burton (2014) showed that even trained passport control officers are not better at matching identities than lay persons, such as university students. The findings reviewed earlier suggest that people with exceptionally good unfamiliar face‐processing skills would be particularly useful in a court of law when it is uncertain whether a defendant's identity matches a person captured on CCTV footage. Current practice in the UK is to employ facial image analysts to evaluate the similarity of the pictorial evidence to the suspect using a number of techniques — a task at which trained experts appear to be better than the general public (Wilkinson & Evans, 2009). However, despite utilising various techniques in face mapping, the current guidelines of the Forensic Imagery Analysis Group state that a positive identification can only be made on the basis of distinctive features such as marks, scars or tattoos (Plews et al., 2004). Further, few attempts to train face‐matching skills in typical perceivers have been reported. Two investigations have not been able to improve face‐matching skills whatsoever (Towler, White & Kemp, 2014; Woodhead, Baddeley & Simmonds, 1979), although a recent study reported promising results that suggest feedback can be a useful training technique (White, Kemp, Jenkins & Burton, 2014). Clearly, much further research is needed to fully develop successful training techniques before they can be implemented within real‐world settings.

An alternative is to employ people who naturally have extraordinary face‐processing skills. These individuals may not only be more adept at making identifications from CCTV footage in a court of law but may also be more proficient at face recognition tasks in national security settings, such as passport control. Pertinently, recent work has identified people with superior face‐processing skills (Russell et al., 2009). These so‐called ‘super‐recognisers’ (SRs) approached the research team believing that they had face‐processing skills that extended beyond those of typical perceivers. They also performed approximately two standard deviations (*SD*) above the control mean on standardised tests of face memory [the Cambridge Face Memory Test long form (CFMT+), Russell et al., 2009] and face perception (Cambridge Face Perception Test, Duchaine, Germine & Nakayama, 2007; cf. Russell, Chatterjee & Nakayama, 2012). Very little is known about the processes underlying such proficient face processing, but it is possible that if SRs perform well on laboratory‐based tests, they would also achieve better results in applied and real‐world tasks involving face matching or face recognition. If this is the case, SRs may be an invaluable resource for forensic and national security agencies.

The current investigation examined whether seven SRs are more proficient than control participants at two face‐processing tasks that resemble real‐world scenarios. In experiment 1, participants had to match still images (taken from video footage) of unfamiliar male faces to arrays containing 10 photographs. Similar circumstances can occur in real‐world scenarios when the face of a perpetrator is captured on CCTV, and a suspect has been apprehended based on their apparent similarity to this person. In experiment 2, participants were required to encode a set of faces and subsequently discriminate them from distractor faces when viewed within brief clips of video footage. Such a scenario could occur when missing or wanted persons are spotted by police officers on patrol. No study to date has investigated the ability of SRs to match faces from video stills to photographs — a task that typical perceivers are remarkably poor at (Bruce et al., 1999). Similarly, while there is strong evidence that typical observers are poor at remembering unfamiliar faces (Burton et al., 1999), there are no data demonstrating the performance of SRs on this type of task. The main aim of this investigation was therefore to establish whether SRs are better at (a) face matching, particularly when the viewpoint of target and line‐up faces differ, and (b) identifying faces from video when a short delay and interference are introduced.

## Experiment 1

In an initial experiment, we investigated the performance of SRs and typical perceivers on a face‐matching task that has been well‐validated in previous research (Bindemann, Brown, Koyas & Russ 2012; Bruce et al., 1999; Megreya, Sandford & Burton, 2013). On each trial, participants were required to match a video still of an unfamiliar male face to an array containing 10 faces. Both target‐present and target‐absent arrays were included, with targets appearing in the arrays on half of the trials. We predicted that SRs would be considerably better at the matching task than typical observers, perhaps because of enhanced encoding strategies and/or more efficient retrieval, and that they would report greater confidence in their responses.

### Method

#### Participants

Seven SRs who were already known to our laboratory participated in this study, and their demographic information is reported in Table 1. These individuals independently contacted our research team because they believed they had superior face recognition skills. As in previous work, super‐recognition was confirmed in these participants using the CFMT+ (Russell, Chatterjee & Nakayama, 2012) — an extension of the well‐used and standardised CFMT (for details, see Duchaine & Nakayama, 2006) that has added difficulty via the inclusion of an extra section that displays heavily degraded faces for recognition. As a group, the seven SRs performed significantly better (*M* = 95.75, *SD* = 2.28) than the published control norm for this test (*M* = 75.20, *SD* = 11.60), *t*(31) = 4.61, *p* < .001, *d* = 1.96, 95% CI [0.98, 2.92], and on par with SRs from previous studies (Russell et al., 2009, 2012; all *p*s > .05; Table 1). Indeed, all SRs outperformed the published control mean by 1.4 to 2.1 *SD*s. All seven SRs were retained in the study based on this performance, as the variability in their performance allowed us to additionally examine whether CFMT score correlates with face‐processing performance on more real‐world tasks.

**Table 1:** Demographical information and CFMT+ scores for the SR participants used in this study and those described by Russell et al. (2012)

	Russell et al. (2012)(*N* = 6)	The current study							
		SRs (*N* = 7)	SR1	SR2	SR3	SR4	SR5	SR6	SR7
Age	40.7 (9.9)	28.7 (7.0)	36	19	37	28	21	23	27
Gender	—	M = 4	M	M	F	M	M	F	F
Hand	—	R = 6	R	R	R	R	R	L	R
CFMT+	95.0 (1.9)	95.71 (1.53)	92	97	97	97	100	93	94

*Note:* Published norms for typical perceivers on this test are also presented by Russell et al. (2012): *M* = 75.20, *SD* = 11.60.

CFMT+, Cambridge Face Memory Test long form; SR, super‐recognisers.

Control participants (*N* = 22) were recruited amongst students and visitors to the University of Stirling. All control participants reported typical face recognition skills, and this was confirmed using the CFMT. However, two participants obtained CFMT scores within the impaired range (a score of 42/72 or below; Duchaine & Nakayama, 2006) and were excluded from all analyses (the CFMT scores of the remaining participants ranged from 44/72 to 69/72). Hence, a total of 20 controls (10 male) were included in this experiment, and their age ranged from 18 to 34 years (*M* = 25.1, *SD* = 6.0). All participants had normal or corrected‐to‐normal vision and participated in exchange for a small monetary payment or course credits. Ethical approval for this study was granted by the University of Stirling Psychology Ethics Committee.

#### Materials

**Figure 1:**
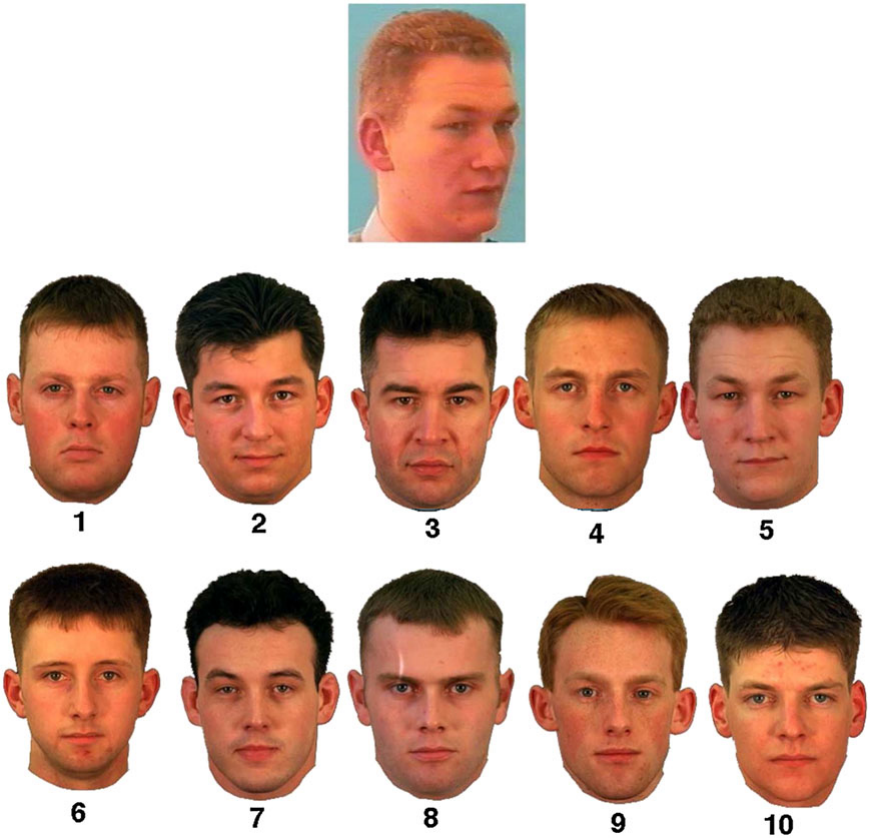
An example trial from a face‐matching array (Bruce et al., 1999; not drawn to scale). The target or probe is a video still. The images are those paired with the target by Bruce et al. (1999). The target is present in position 5

This experiment used the same stimuli that were developed by Bruce et al. (1999). Each trial consisted of a still image extracted from video footage, displaying a male face from a 30° viewpoint. The target image was simultaneously presented above an array of 10 male faces depicted from full face viewpoints and arranged in a 5 × 2 line‐up. All images were displayed in colour. On half of the trials, the 10 photographs resembled but did not contain the target image (target‐absent condition), and on the other half, the photograph of the target was amongst the 10 photographs (target‐present condition). Photographs in the arrays were numbered 1–10, and the position of the target varied with the constraint that each position was used four times. These photographs were cropped so that no clothing was displayed (but the external facial features including the hair were still visible) and were arranged in arrays of 1050 × 700 pixels. Target video stills were cropped below the shoulders so that some clothing was still visible. These images measured 216 pixels (*W*) × 263 pixels (*H*). An example trial array is presented in Figure 1.

#### Design and procedure

A 2 × 2 mixed factorial design was employed. The within‐subjects factor was target presence (target present/target absent), and the between‐subjects factor was participant group (controls/SRs). Each individual saw all 80 arrays, with the target present on half of the trials. Trials were presented in a fully randomised order.

All participants were tested individually using e‐prime software (Psychology Software Tools, Sharpsburg, PA, USA) and a 15.6‐inch liquid‐crystal display (LCD) monitor displayed at a resolution of 1366 × 768 pixels. Participants sat at a comfortable distance from the screen and gave their responses using keys on a keyboard under no time constraints. They were instructed that they were going to complete a face‐matching task and that the target may or may not be present in each line‐up. Targets were identified using the corresponding keyboard number (for targets identified as present in location 10, participants were instructed to press 0). For arrays where no match was detected, participants were told to press the space bar. After each array, participants rated their confidence in their response on a scale from 1 (*not at all confident*) to 5 (*very confident*).

#### Statistical analyses

The percentages of hits (correct identifications in target‐present arrays), misses (no‐match decisions in target‐present trials), false identifications (selection of a non‐match face in target‐present trials), correct rejections (correct target‐absent responses in target‐absent arrays) and false‐positive responses (false identification of a face in target‐absent trials) were calculated for each participant.

While accuracy is a good indicator of the overall patterns of responses between the SR and control groups, additional analyses regarding sensitivity and response criterion permit more in‐depth understanding of the differences between them and are useful for cross‐study comparison (McIntyre, Hancock, Kittler & Langton, 2013). In line‐up paradigms with target‐present and target‐absent arrays, there are five types of response (discussed in the preceding text), which is not typical for signal detection analyses. We therefore only used correct responses from target‐present trials (hits) and false‐positive responses from target‐absent trials in these comparisons. Specifically, *d* prime (*d*′), a measure of sensitivity, was calculated by subtracting the *z* scores for false‐positive (F) responses in the target‐absent trials from *z* scores calculated from correct identifications (hits, H) in target‐present trials [*d*′ = *z*(H) − *z*(F)] (Table 2). Response bias (criterion *c*) was calculated as the negative average sum of *z* scores for the hits and false‐positive response *c* = −0.5[*z*(H) + *z*(FPs)] (Macmillan & Creelman, 2004).

**Table 2:** Mean (*SD*) accuracy score for SRs and control participants in experiment 1

	Target present (%)			Target absent (%)		
	Hits	Miss	False ID	Correct rejection	*d*′	c
SRs	93.57 (4.75)	1.07 (1.33)	5.36 (4.61)	92.14 (7.69)	3.20 (0.59)	−0.01 (0.37)
Controls	80.87 (9.07)	9.75 (8.26)	9.37 (6.17)	65.12 (21.98)	1.42 (0.90)	−0.25 (0.30)

*Note:* Signal detection scores are calculated from the *z* scores of hits and false‐positive identifications from target‐absent arrays.

*SD*, standard deviation; SRs, super‐recognisers.

### Results

#### Accuracy

A 2 × 2 repeated‐measures analysis of variance (ANOVA) was conducted on accuracy scores, with array type as the within‐participant factor (target present/target absent) and group as the between‐participant factor (SRs/controls). A significant main effect of array type indicated that performance was more accurate for target present [*M* = 87.2%, standard error (*SE*) = 1.8%] than for target absent (*M* = 78.6%, *SE* = 4.3) trials, *F*(1, 25) = 4.54, *p* = .043, η_p_^2^ = .15, 95% CI [0.00, 0.39]. There was also a significant main effect of group with a higher percentage of correct responses being made by the SRs (*M* = 92.90%, *SE* = 4.60) than controls (*M* = 73.00%, *SE* = 2.70), *F*(1, 25) = 14.55, *p* = .001, η_p_^2^ = .368, 95% CI [0.08, 0.57]. Although the interaction between array type and participant group was not statistically significant, this may be due to the small number of participants in the SR group, *F*(1, 25) = 3.16, *p* = .088, η_p_^2^ = .112, 95% CI [0.00, 0.35] (Table 2). Indeed, independent analyses of group differences on target‐present and target‐absent trials revealed that SRs performed significantly better than controls regardless of array type, *t*(25) = 3.50, *p* = .002, *d* = 1.37, 95% CI [0.57, 2.49] and *t*(25) = 3.13, *p* = .004, *d* = 1.37, 95% CI [0.42, 2.30], respectively.

Analyses of mistakes on target‐present trials (false IDs, i.e. choosing the wrong face in target‐present arrays and misses, errors on target‐present arrays where participants responded ‘no match’) were conducted using a 2 × 2 repeated‐measures ANOVA with mistake type as the within‐participant factor (miss/false ID) and group as the between‐participant factor (SRs/controls). SRs made fewer mistakes overall on target‐present trials (*M* = 3.20%, *SE* = 0.09%) than controls (*M* = 9.60%, *SE* = 1.60%), *F*(1, 25) = 12.28, *p* = .002, η_p_^2^ = .329, 95% CI[0.06, 0.54]. The main effect of mistake type and the interaction did not reach statistical significance, *F*(1, 25) = 0.75, *p* = .393, η_p_^2^ = .029, 95% CI [0.00, 23] and *F*(1, 25) = 1.07, *p* = .310, η_p_^2^ = .041, 95% CI [0.00, 25] respectively.

#### Signal detection analyses

An independent‐samples *t*‐test on *d*′ scores revealed higher sensitivity in SRs than controls, *t*(25) = 5.15, *p* < .001, *d* = 2.45, 95% CI [1.18, 3.30] (Table 2). A *t*‐test on criterion *c* scores revealed a non‐significant difference between the groups *t*(25) = 1.48, *p* = .151, *d* = 0.64, 95% CI [−0.23, 1.52]. In sum, these findings indicate, in line with accuracy analyses, that SRs are better at unfamiliar face discrimination in a matching task. On an individual level, we performed modified *t*‐tests for single‐case comparisons (Crawford, Garthwaite & Porter, 2010) on the *d*′ and criterion *c* data for each of the SRs in comparison with controls. Four participants, SR1, SR3, SR4 and SR7, showed significantly higher sensitivity than controls on the matching task. The response bias of SR7 was also more conservative than that of the control group. Full results are presented in Table 3.

**Table 3:** Individual case analyses of sensitivity of SRs in experiment 1, using modified *t*‐tests for single‐case comparisons (Crawford et al., 2010)

	Control mean (*SD*) (*N* = 20)	SR1	SR2	SR3	SR4	SR5	SR6	SR7
*d*′	1.39 (0.85)	3.92	2.37	3.24	3.92	3.11	2.58	3.28
*t*(19)	—	2.90	1.12	2.12	2.90	1.97	1.37	2.17
*p* (two‐tailed)	—	.009	.274	.047	.009	.063	.188	.043
*Zcc*	—	2.98	1.15	2.18	2.98	2.02	1.40	2.22
95% CI for *Zcc*	—	[1.93, 4.00]	[0.57, 1.71]	[1.35, 2.98]	[1.93, 4.00]	[1.24, 2.78]	[0.77, 2.01]	[1.39, 3.04]
Population below individual's score (%)	—	99.54	86.27	97.64	99.54	96.85	90.60	97.85
Criterion *c*	−0.23 (0.32)	0.00	−0.25	0.33	0.00	−0.40	−0.35	0.60
*t*(19)	—	0.69	−0.07	1.71	0.69	−0.53	−0.38	2.50
*p* (two‐tailed)	—	.499	.945	.104	.499	.604	.710	.021
*Zcc*	—	0.71	−0.01	1.75	0.71	−0.54	−0.39	2.56
95% CI for *Zcc*	—	[0.21, 1.19]	[−0.51, 0.36]	[1.03, 2.44]	[0.21, 1.19]	[−1.00, 0.06]	[−0.84, 0.07]	[1.63, 3.47]
Population below individual's score (%)	—	75.03	47.26	94.79	75.03	30.21	35.49	98.91

*Note: SD*, standard deviation; SRs, super‐recognisers.

#### Confidence

Overall, SRs were more confident (*M* = 4.29, *SD* = 0.40) in their responses than control participants (*M* = 3.69, *SD* = 0.36), regardless of the trial type or actual accuracy *t*(25) = 3.63, *p* = .001, *d* = 1.38, 95% CI [0.63, 2.53]. Mean confidence scores for correct responses were analysed using a 2 (response type: hit/correct rejection) × 2 (groups: SRs/controls) mixed factorial ANOVA. The analysis revealed a significant main effect of response type, *F*(1, 25) = 30.73, *p* < .001, η_p_^2^ = .551, 95% CI [0.25, 0.70] with participants reporting higher confidence following correct responses in target‐present (*M* = 4.36, *SE* = 0.08) than target‐absent (*M* = 3.78, *SE* = 0.11) trials. There was also a significant main effect of group *F*(1, 25) = 10.41, *p* = .003, η_p_^2^ = .294, 95% CI [0.04, 0.51] with SRs reporting higher confidence for correct responses (*M* = 4.33, *SE* = 0.14) than controls (*M* = 3.80, *SE* = 0.08). The interaction between response type and group was non‐significant, *F*(1, 25) = 2.92, *p* = .100, η_p_^2^ = .104, 95% CI [0.00, 0.34]. As some participants did not commit each type of error, confidence for incorrect responses was analysed separately for misses, false IDs and FPs using independent‐samples *t*‐tests. Participants whose data were missing were not included in analyses involving these mistakes. The differences between groups on confidence ratings following incorrect responses did not reach statistical significance, all *p*s > .05.

#### CFMT and matching‐task performance

**Figure 2:**
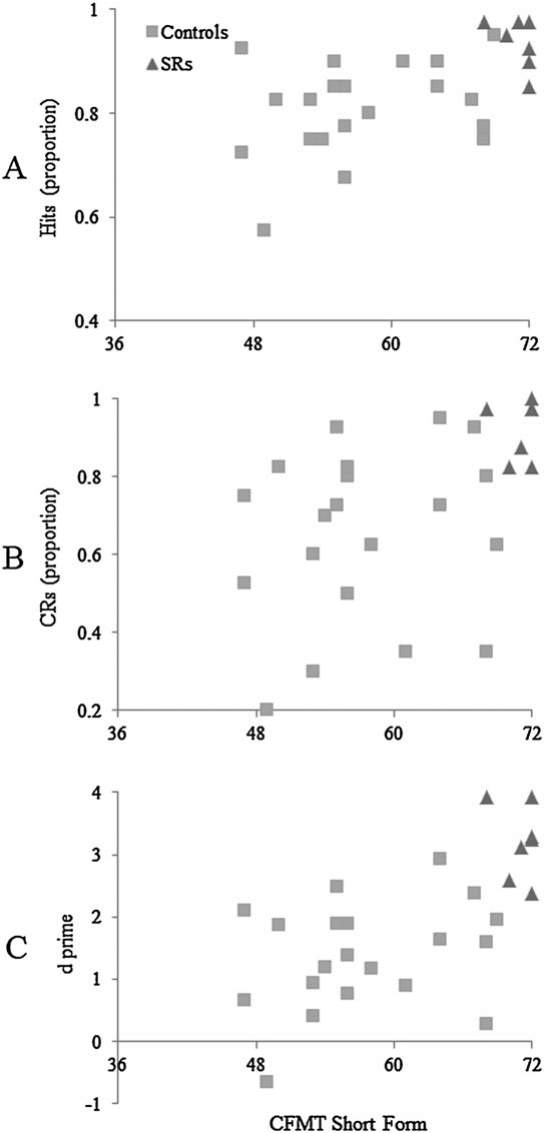
Proportion of hits (A), CRs (B) and *d*′ (C) on the 1‐in‐10 task in experiment 1 plotted against Cambridge Face Memory Test (CFMT) score. The dependent variable in the CFMT is the number of correct responses from a maximum score of 72. SRs, super‐recognisers

Because CFMT scores were available for all our participants (controls were initially screened using the CFMT, and performance on the shorted form of the test can be calculated from the SRs' CFMT+ scores), we correlated performance on this task with the matching test. Performance on the CFMT and sensitivity on the 1‐in‐10 matching task is presented in Figure 2. Each marker represents a separate data point. Notably, performance on the CFMT predicted performance on the matching task, according to both the proportion of hits, *N* = 27, Spearman'*s ρ* = .606, *p* = .001, 95% CI [0.29, 0.80] correct rejections, *N* = 27, Spearman's *ρ* = .566, *p* = .002, 95% CI [0.24, 0.78] and *d*′, *N* = 27, Spearman's *ρ* = .633, *p* < .001, 95% CI [0.33, 0.82].

### Discussion

This experiment investigated the performance of SRs and typical perceivers on a face‐matching task consisting of target‐present and target‐absent line‐ups. Our prediction that SRs would outperform controls on this task was supported: SRs were better at the task both in terms of accuracy and perceptual sensitivity measures. SRs were also more confident in their responses than controls in overall analyses but did not differ from controls when making a ‘no‐match response’ on target‐absent trials. The overall advantage in confidence for SRs may arise from self‐awareness of their above‐average face‐processing skills. On an individual level, four SRs performed significantly better than the control group, and one SR was more conservative in their response bias than controls.

Interestingly, for both control and SR participants, CFMT performance reliably predicted face‐matching performance on target‐present and target‐absent trials, and the *d*′ measure. Similar results were reported in a recent study by White et al. (2014). The authors found that performance on the Glasgow Face Matching Test (Burton, White and McNeill, 2010) was a good predictor of performance on a photograph‐to‐person matching task, although this finding only emerged for mismatched trials given there were ceiling effects on matched trials. Our study extends these findings by showing that the CFMT, a standardised test widely used in face recognition research, might also be useful for forensic practice. However, the results reported here suggest that CFMT should be supplemented with applied tasks in order to gain full understanding of an individual's performance that could be generalised to real‐world settings.

## Experiment 2

In experiment 2, we examined face memory in SRs compared with typical perceivers. In the study phase, all participants were presented with 20 good quality stills of male and female faces displayed from a frontal viewpoint. After a letter search filler task, participants viewed 40 video clips where targets were present on half of the trials. In certain situations (such as the 2011 England riots), multiple people are sought by the police, increasing memory load for unfamiliar faces. Diligent officers may also study ‘wanted’ posters, with a view to looking out for the individuals concerned. We predicted that SRs would perform considerably better on this task and report higher confidence ratings in their responses than control participants.

### Method

#### Participants

The same seven SRs as described in the preceding text took part in experiment 2 (Table 1). Twenty control participants (10 male) were also recruited amongst students and visitors to the University of Stirling. Their ages ranged from 19 to 33 years (*M* = 24.4, *SD* = 5.7). As for experiment 1, all control participants were screened using the CFMT (Duchaine & Nakayama, 2006) in order to exclude those potentially affected by prosopagnosia or meeting the criteria for super‐recognition. No participants were excluded from the control sample, however. The CFMT scores of control participants ranged from 47/72 to 69/72. All controls participated for course credit or a small monetary payment. The study was granted ethical approval by the University of Stirling Psychology Ethics Committee.

#### Materials

**Figure 3:**
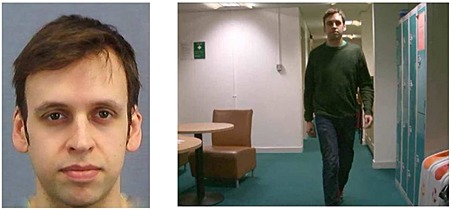
Example stimuli from study and test (not drawn to scale) in experiment 2. The target is present in the video clip on the right

Twenty good quality facial images (10 male) extracted from video footage were taken from the Psychological Image Collection at Stirling (http://pics.stir.ac.uk/). The stills measured 390 pixels (*W*) × 480 pixels (*H*) and were cropped below the neck. They were not converted to grayscale to mimic the natural settings when a search for a missing or wanted person would occur. All visible clothing and jewellery was removed using Adobe Photoshop (Adobe Systems, San Jose, California, US). Forty video clips (20 containing the person depicted in the still images) were extracted from the same database. The clips were sized 640 pixels (*W*) × 480 pixels (*H*) and played at frame rate of 25 frames per second. All videos were adjusted to a duration of 5 seconds using magix movie edit pro (Magix Software GmBH, Berlin, Germany). Each clip depicted individuals walking down a corridor in dim lighting, and half were male. Example stimuli from the study and test phases are presented in Figure 3.

#### Design and procedure

A 2 (target presence: present, absent) × 2 (participant group: SRs, controls) mixed factorial design was employed. All participants were tested individually using e‐prime software (Psychology Software Tools) with a 15.6‐inch LCD monitor displayed at a resolution of 1366 × 768 pixels. They sat at a comfortable distance from the screen and responded using the keyboard. In the study phase, participants were instructed that they should view the 20 individuals carefully as if they were missing or wanted. The stills were presented in a random order for 5 seconds each. Participants then took part in a simple letter search filler task, which was followed by a break, so that the total time between the study and test phases was 20 minutes. At test, participants were instructed to view the 40 clips (presented in a random order) and indicate whether the person in the video was familiar or not using the *M* or *Z* keyboard keys (response mapping was counterbalanced between participants). The duration of all clips was 5 seconds, and if participants did not respond during that time, the experiment proceeded to a response screen where participants were prompted to make their response. After each trial, participants were asked to rate their confidence in their response on a scale of 1 (*not at all confident*) to 5 (*very confident*).

#### Statistical analyses

The percentage of hits (correct identifications in target‐present trials) and correct rejections (target‐absent trials that were responded to as such) was calculated for each participant. The discrimination of identities appearing in the video clips was also analysed using the signal detection measures of sensitivity and response bias.

### Results

#### Accuracy

A 2 (target‐presence: target present, target absent) × 2 (participant group: SRs, controls) mixed factorial ANOVA was conducted on accuracy scores. A significant main effect of group indicated that SRs (*M* = 67.00%, *SE* = 2.80) were better at this task than control participants (*M* = 58.60%, *SE* = 1.80), *F*(1, 25) = 5.68, *p* = .025, η_p_^2^ = .185, 95% CI [0.02, 0.42] (Table 4). Neither the main effect of clip type (i.e. target present versus target absent) nor the interaction with participant group reached significance, *F*(1, 25) = 0.57, *p* = .455, η_p_^2^ = .022, 95% CI [0.00, 0.21] and *F*(1, 25) = 0.19, *p* = .666, η_p_^2^ = .008, 95% CI [0.00, 17], respectively.

**Table 4:** Performance of SR and control participants in experiment 2

	Hits (%)	Correct rejections (%)	*d*′	*c*
SRs	64.64 (20.12)	69.29 (9.32)	1.00 (0.59)	0.02 (0.48)
Controls	58.00 (9.65)	59.25 (11.15)	0.45 (0.42)	0.01 (0.18)

*Note:* Sensitivity and response bias are calculated from the *z* scores of hits and false‐positive identifications from target‐absent trials.

SRs, super‐recognisers.

#### Signal detection analyses

An independent‐samples *t*‐test on *d*′ scores indicated that SRs had greater sensitivity than controls, *t*(25) = 2.64, *p* = .01, *d* = 1.33, 95% CI [0.23, 2.06] (Table 4). However, the equivalent analysis of response bias (criterion *c*) yielded no significant differences between groups, *t*(25) = 0.06, *p* = .995, *d* = 0.03, 95% CI [−0.83, 0.89]. Taken together, signal detection analyses indicate that SRs are better than typical perceivers at discriminating facial identity from poor‐quality video clips. In line with experiment 1, we performed case‐by‐case analyses on *d*′ and criterion *c* for SRs and control participants. Full results are presented in Table 5.

**Table 5:** Individual case analyses of sensitivity and response bias of SRs in experiment 2, using modified *t*‐tests for single‐case comparisons (Crawford et al., 2010)

	Control mean (*SD*) (*N* = 20)	SR1	SR2	SR3	SR4	SR5	SR6	SR7
*d*′	0.45 (0.45)	0.64	0.32	0.77	2.09	1.23	0.64	1.35
*t*(19)	—	0.40	−0.29	0.69	3.55	1.73	0.40	1.95
*p* (two‐tailed)	—	.690	.774	.500	.002	.108	.690	.067
*Zcc*	—	0.41	−0.30	0.70	3.64	1.73	0.41	1.99
95% CI for *Zcc*	—	[−0.05, 0.86]	[−0.74, 0.15]	[0.20, 1.19]	[1.02, 2.42]	[1.24, 2.78]	[−0.05, 0.86]	[1.22, 2.75]
Population below individual's score (%)	—	65.48	38.74	74.97	99.89	96.85	65.48	96.67
Criterion *c*	0.02 (0.18)	0.07	−0.25	0.00	−0.92	0.23	0.07	0.00
*t*(19)	—	0.29	−0.07	−0.09	−5.12	−0.53	0.29	−0.09
*p* (two‐tailed)	—	.777	.945	.925	<.001	.604	.777	.925
*Zcc*	—	0.29	−0.01	−0.10	−5.24	−0.54	0.29	−0.09
95% CI for *Zcc*	—	[−0.16, 0.74]	[−0.51, 0.36]	[−0.53, 0.34]	[−6.94, −3.53]	[−1.00, 0.06]	[−0.16, 0.74]	[−0.53, 0.34]
Population below individual's score (%)	—	61.16	47.26	0.003	75.03	30.21	61.16	46.29

*Note: SD*, standard deviation; SRs, super‐recognisers.

#### Confidence

There was no difference in overall confidence ratings between groups, *t*(25) = 0.17, *p* = .861, *d* = 0.07 95% CI [−0.79, 0.93]. Mean confidence scores for hits and correct rejections were analysed using a 2 × 2 ANOVA with one between‐participants (group: SRs/controls) and one within‐participants (response type: hits/correct rejections) factor. The analysis revealed a significant main effect of trial type, with higher confidence reported for correct responses when a target was present in the clip (hits, *M* = 3.80, *SE* = 0.11) than for when participants correctly rejected a video without a target (correct rejections, *M* = 3.15, *SE* = 0.14), *F*(1, 25) = 49.83, *p* < .001, η_p_^2^ = .666, 95% CI [0.40, 0.78]. The main effect was qualified by a significant interaction between the factor trial type and group, *F*(1, 25) = 8.96, *p* = .006, η_p_^2^ = .264, 95% CI [0.02, 0.49]. The main effect of group did not reach statistical significance, *F*(1, 25) = .002, *p* = .964, η_p_^2^ = .00008 95% CI [0.00, 0.00]. The interaction was explored using independent sample *t*‐test for each level of trial type. The analysis, however, revealed no significant differences between groups in confidence ratings for either type of trial (*p*s > .05), reinforcing the non‐significant main effect of group in the ANOVA results. For the purpose of analysis of erroneous trials (misses and FPs), one SR who correctly rejected all target‐absent clips and therefore did not make any FPs was removed from this analysis. A 2 (target presence: target present, target absent) × 2 (participant group: SRs, controls) mixed factorial ANOVA revealed higher confidence levels for FPs (*M* = 3.39, *SE* = 0.13) than misses (*M* = 2.90, *SE* = 0.13), *F*(1, 24) = 13.38, *p* = .001, η_p_^2^ = .358, 95% CI [0.07, 0.57]. There were no significant differences between groups, and the interaction did not reach statistical significance, *F*(1, 24) = 0.65, *p* = .428, η_p_^2^ = .026, 95% CI [0.00, 0.23] and *F*(1, 24) = 0.31, *p* = .582, η_p_^2^ = .013, 95% CI [0.00, 19].

#### CFMT and face memory scores

**Figure 4:**
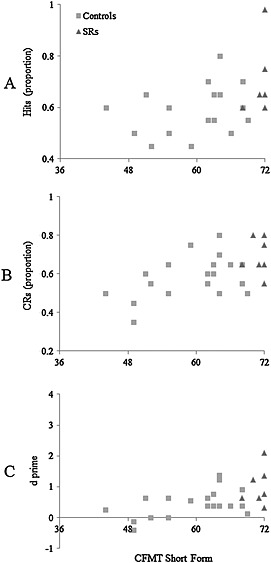
Proportion of hits (A), CRs (B) and *d*′ (C) on the face memory task in experiment 2 plotted against Cambridge Face Memory Test (CFMT) score. The dependent variable in the CFMT is the number correct from a maximum score of 72. SRs, super‐recognisers

As for experiment 1, we correlated the CFMT scores of all participants (controls and SRs) with the proportion of hits and CRs, and *d*′ scores, that they achieved on this task (Figure 4). Once more, CFMT score was a good predictor of performance on target‐present trials, *N* = 27, Spearman's *ρ* = .383, *p* = .048, 95% CI [0.00, 0.67], target‐absent trials, *N* = 27, Spearman's *ρ* = .462, *p* = .015, 95% CI [0.10, 0.72] and *d*′, *N* = 27, Spearman's *ρ* = .531, *p* = .004, 95% CI [0.19, 0.76].

### Discussion

Experiment 2 investigated whether SRs outperform typical perceivers on an applied test of unfamiliar face memory. It has been previously reported that the performance of the general population on this type of test is remarkably poor and close to chance level (Burton et al., 1999). We predicted that SRs would be better than control participants at recognising unfamiliar faces from poor‐quality video clips, perhaps because of enhanced encoding and more efficient retrieval upon presentation. Findings from this experiment show that SRs are better than control participants at this task and suggest that they may be useful in applied forensic and security settings. It is noteworthy, however, that the overall accuracy of the SR group was relatively low, with approximately 35% of trials resulting in ‘misses’ and 31% in FPs. On the other hand, control participants performed marginally, but significantly above the chance level on each type of trial, replicating the findings of previous work (e.g. Burton et al., 1999). The low accuracy rate occurred despite the video footage being of relatively high quality. Surveillance systems relying on older technology may produce poorer quality footage recorded from a less favourable viewpoint. The results of this study may therefore be only applicable to higher quality CCTV systems. Interestingly, while recognition performance was better in the SR group, confidence ratings were not. This may be explained by the high level of difficulty of the task and the brief presentation of stimuli in both the study and test phases. Nevertheless, SRs were still able to outperform typical perceivers, suggesting they may be valuable employees in forensic and security settings. On an individual level, SR4 was the only participant who was significantly better than controls at discriminating between identities in the test phase. Notably, he also showed an increased bias towards making a positive familiarity decision when a target was absent.

Finally, the CFMT was again found to be a good predictor of performance on this task, although the correlation was weaker than that calculated for the matching task in experiment 1. It is likely that this difference is underlined by the relative difficulty of experiment 2, where many control participants performed at near‐chance levels.

## General Discussion

This investigation set out to examine the performance of seven SRs on a face‐matching and a face memory task. In experiment 1, we compared the performance of SRs to typical perceivers on a well‐established 1‐in‐10 unfamiliar face‐matching task. In experiment 2, we tested SRs' memory for faces encoded from high‐quality CCTV stills that were later presented in poor‐quality video clips. In both studies, we also measured participants' confidence in their responses. Finally, we correlated a standardised test of face memory (the CFMT: Duchaine & Nakayama, 2006) with the overall performance of all participants in experiments 1 and 2. As predicted, SRs outperformed controls in both experiments but were only more confident in their performance in the face‐matching task. Further, CFMT performance was found to be a good predictor of performance on both the face‐matching and face recognition tasks.

There are a number of theoretical and practical implications that arise from this pattern of findings. In terms of theory, research examining unfamiliar face processing has long been concerned with viewpoint and lighting change between study and test images. For instance, Longmore, Liu and Young (2008) reported that change in the direction of illumination and pose between study and test leads to reduced face recognition performance. In matching studies, where there is no memory load in the task or the load is minimal, viewpoint and lighting changes also lead to decrements in performance (Braje, 2003; Bruce et al., 1999). In our study, the arrays used in experiment 1 were taken from the stimuli set developed by Bruce et al. (1999). We used the most difficult variant of the task, where target and line‐up photographs differed according to viewpoint, and different cameras were used to capture each image. According to the Bruce and Young (1986) model of face recognition, a change in viewpoint should activate structural rather than pictorial encoding strategies, so the invariant parts of the facial image are encoded. While participants in the control group performed on par with the original line‐up study where the reported error rate was approximately 40% (with the exception of the target‐present condition in experiment 1 where accuracy was higher), SRs were significantly better at face matching with mistakes being made on less than 8% of the trials in both conditions. This finding suggests that SRs may have better mechanisms for the structural encoding of faces than typical perceivers. A more recent cognitive neuroscience model developed by Haxby, Hoffman and Gobbini (2000, 2002) proposes that static components of a facial image, such as a view‐independent representation, are analysed via a route involving the lateral fusiform gyrus. It is possible that this neural mechanism is better developed in SRs, whereas typical perceivers rely heavily on pictorial representations, prone to disruption by viewpoint changes. Another possibility is that SRs are more proficient at manipulating pictorial codes than typical perceivers. A study using un‐textured three‐dimensional faces to investigate performance of SRs would elucidate this phenomenon (e.g. Bruce & Langton, 1994).

However, the results are somewhat less clear cut in experiment 2. While the overall accuracy performance of the SR group was higher than that of the control group, overall performance was generally poor with error rates of 33% and 42% for SR and control participants, respectively. It is possible that this is due to the particular paradigm we employed. Participants in our study performed a letter search task for 15 minutes and had a short break of 5 minutes between study and test. This was in order to introduce some distractions that may occur in real life while not requiring participants to participate on two separate occasions and retaining a degree of experimental control. Yet, the filler task required local letter recognition and was performed directly before recall of the encoded faces. This may have interfered with the newly learned faces by promoting a suboptimal featural processing strategy (Lewis, Mills, Hills & Weston, 2009; cf. Farah, 1991). Nonetheless, albeit prone to errors, performance of the SRs was remarkably better than that of control participants, despite different image quality, resolution, size and lighting between the study and test phases. However, it is important to note that while all the aforementioned variables were different, viewpoint remained unchanged between the study and test aside from individual variations in head tilt in the test phase (the individuals presented in the clips walked for 5 seconds along a dimly lit corridor). It is possible that participants were trying to activate pictorial codes in order to reach a decision, but the video clips were insufficient to match a stored image of a studied face against one presented at test. SRs, as argued before, may have developed a more efficient mechanism for structural encoding and thus are able to form more stable representations of studied faces that facilitate recognition under unfavourable conditions.

The implications for forensic practice are also important to note. Existing evidence suggests that (a) people are poor at unfamiliar face matching (Bruce et al., 1999; Kemp et al., 1997; Megreya et al., 2013) and recognition (Burton et al., 1999), (b) the results of training in face matching are limited (Towler et al., 2014; Woodhead et al., 1979; cf. Kemp et al., 2014) and (c) the face‐matching ability of employees in security settings is unrelated to years of service and, ultimately, experience (White et al., 2014). Our study clearly shows that SRs are significantly better at face matching (experiment 1) and recognition (experiment 2) than a sample of typical observers. Results from both experiments also show that performance on a standardised test of face recognition (the CFMT: Duchaine & Nakayama, 2006) is related to accuracy in face matching and recognition. Current training studies often use shape classification to improve face recognition, a method which has had little success to date (Towler et al., 2014). A possible way of improving training programmes is to apply findings from detailed investigations of the processes underlying extraordinary face recognition — a study that is currently underway in our laboratory. Another, more readily available, solution would be to select specific personnel for positions requiring excellent face‐processing skills based on standardised aptitude tests, such as the CFMT. Burton, White and McNeill (2010) showed that the Glasgow Face Matching Test, but not a simple two‐alternative forced‐choice face memory test, predicts face‐matching performance. Our results seem to contradict this, with the CFMT emerging as a good predictor of both face‐matching and recognition accuracy. This discrepancy is most likely due to methodological differences between the CFMT and the memory task used in the study by Burton et al. (2010). The CFMT gradually familiarises participants with multiple faces and introduces different viewpoints at study and test making on‐line feature matching impossible. It is thus a more sophisticated tool for assessing both memory and perception of faces that can predict complex tasks of face matching and recognition. The standard version of the CFMT only takes approximately 10 minutes to administer and can thus be a valuable addition for employee selection processes in security settings. What is more, we recommend similar practice in forensic investigations and practice. Our study shows that SRs are significantly better at face matching, and recognition — skills that are essential for police officers or personnel working with CCTV footage. The Metropolitan Police currently employ SR officers, identified within the Force by the means of positive identifications leading to arrests of suspects. They are often assigned to specific tasks involving CCTV footage, but to the authors' best knowledge, screening of face‐processing skills is not a standard practice at the time of recruitment. Such aptitude tests should perhaps become a standard part of the enrolment process for all police officers. This would ensure optimal personnel allocation and, when operated by people with good face processing skills, maximise the utility of CCTV systems.

However, it is important to note that while SRs consistently outperformed control participants as a group, the same was not true in all analyses on an individual level. In experiment 1, the discriminability of four SRs was significantly better than that of controls, and one SR showed a more conservative response bias. In experiment 2, one SR performed significantly better than the control group and was also more liberal in their responses. However, another SR performed below the control mean. These single‐case statistics are a particular strength of the work presented here and show that there is a degree of performance variation within the SR population. These differences may be due to heterogeneity in the cognitive and perceptual processes underpinning superior face recognition, as reflected by the varying performance in experiments 1 and 2. Previous research with individuals affected by developmental prosopagnosia (face blindness) shows that even within the same family, deficits associated with impaired face processing can vary between relatives (see Susilo & Duchaine, 2013 for a review). Specific processes underlying expert face recognition are still largely unknown, but it is possible that a similar heterogeneity in the SR group is responsible for some, but not all SRs excelling in different types of applied tasks. It is of particular interest, that within the SR group, CFMT+ performance did not appear to correspond directly to participants' discriminability in the applied tasks. For instance, SR1, who obtained the lowest CFMT+ score in the group (92/102), was amongst the highest performers on the face‐matching task in experiment 1 and was one of the only participants to significantly outperform controls in case‐by‐case analyses. Conversely, SR5, who achieved the highest CFMT+ score in the group (100/102), did not discriminate or remember faces significantly better than controls on an individual level. Similarly, SR7, who scored 94/102 on the CFMT+, outperformed the three SRs with the highest CFMT+ scores (SR1, SR2 and SR5) in experiments 1 and 2. Our data therefore suggest that while SRs as a group are significantly better at face matching and memory than typical perceivers, there is some variability in how well they perform these applied tasks. One possibility is that individual differences in general visual processing could be underlying those differences in performance on applied tasks. Indeed, there is some evidence that developmental prosopagnosia is often underpinned by various deficits in the domain of perception (e.g. De Renzi, Faglioni, Grossi & Nichelli, 1991) and a similar investigation in our laboratory suggests that this is also true for SRs (Bobak, Bennetts, Jansari, Parris & Bate, in preparation). Future work could consider individual differences in general visual processing and performance on applied tasks of face matching and face recognition. Thus, in forensic and national security settings, it would be beneficial to follow up initial screening (i.e. using tests such as the CFMT or CFMT+) with tests resembling real‐life scenarios (such as those involving face matching or memory for faces taken from CCTV footage) in order to ensure optimal personnel allocation.

Taken together, the results from our study show that (i) SRs as a group are sizeably better at applied tasks of face matching and recognition and (ii) the CFMT+ supplied by applied tasks are useful tools for identifying high‐performing individuals with excellent face recognition and matching skills. Research in unfamiliar face processing consistently shows that there are large individual differences in face memory and perception (Bowles et al., 2009; Russell et al., 2009), such that some people experience everyday problems with face recognition (for a review, see Susilo & Duchaine, 2013) while others are particularly good at face processing and claim to ‘never forget a face’ (Russell et al., 2009; Russell et al., 2012). The lack of standardised tests of face recognition in security and forensic settings makes these agencies vulnerable to typical error‐prone performance, and these agencies may benefit from the employment of SRs. These individuals could be used to offer expert opinion for work assignments involving CCTV footage and may be a valuable addition to border control and police personnel.
